# Reducing Firearm Access for Suicide Prevention: Implementation Evaluation of the Web-Based “Lock to Live” Decision Aid in Routine Health Care Encounters

**DOI:** 10.2196/48007

**Published:** 2024-04-22

**Authors:** Julie Angerhofer Richards, Elena Kuo, Christine Stewart, Lisa Shulman, Rebecca Parrish, Ursula Whiteside, Jennifer M Boggs, Gregory E Simon, Ali Rowhani-Rahbar, Marian E Betz

**Affiliations:** 1 Kaiser Permanente Washington Health Research Institute, Seattle, WA, United States; 2 Department of Health Systems and Population Health, University of Washington, Seattle, WA, United States; 3 Department of Mental Health & Wellness, Kaiser Permanente Washington, Seattle, WA, United States; 4 NowMattersNow.org, Seattle, WA, United States; 5 Department of Psychiatry and Behavioral Sciences, University of Washington, Seattle, WA, United States; 6 Kaiser Permanente Colorado Institute for Health Research, Aurora, CO, United States; 7 Department of Epidemiology, School of Public Health, University of Washington, Seattle, WA, United States; 8 Firearm Injury and Policy Research Program, University of Washington, Seattle, WA, United States; 9 Department of Emergency Medicine, University of Colorado School of Medicine, Aurora, CO, United States

**Keywords:** suicide prevention, firearm, internet, implementation, suicide, prevention, decision aid, risk, feasible, support, evaluation, mental health, electronic health record, tool

## Abstract

**Background:**

“Lock to Live” (L2L) is a novel web-based decision aid for helping people at risk of suicide reduce access to firearms. Researchers have demonstrated that L2L is feasible to use and acceptable to patients, but little is known about how to implement L2L during web-based mental health care and in-person contact with clinicians.

**Objective:**

The goal of this project was to support the implementation and evaluation of L2L during routine primary care and mental health specialty web-based and in-person encounters.

**Methods:**

The L2L implementation and evaluation took place at Kaiser Permanente Washington (KPWA)—a large, regional, nonprofit health care system. Three dimensions from the RE-AIM (Reach, Effectiveness, Adoption, Implementation, Maintenance) model*—Reach*, *Adoption*, and *Implementation*—were selected to inform and evaluate the implementation of L2L at KPWA (January 1, 2020, to December 31, 2021). Electronic health record (EHR) data were used to purposefully recruit adult patients, including firearm owners and patients reporting suicidality, to participate in semistructured interviews. Interview themes were used to facilitate L2L implementation and inform subsequent semistructured interviews with clinicians responsible for suicide risk mitigation. Audio-recorded interviews were conducted via the web, transcribed, and coded, using a rapid qualitative inquiry approach. A descriptive analysis of EHR data was performed to summarize L2L reach and adoption among patients identified at high risk of suicide.

**Results:**

The initial implementation consisted of updates for clinicians to add a URL and QR code referencing L2L to the safety planning EHR templates. Recommendations about introducing L2L were subsequently derived from the thematic analysis of semistructured interviews with patients (n=36), which included (1) “have an open conversation,” (2) “validate their situation,” (3) “share what to expect,” (4) “make it accessible and memorable,” and (5) “walk through the tool.” Clinicians’ interviews (n=30) showed a strong preference to have L2L included by default in the EHR-based safety planning template (in contrast to adding it manually). During the 2-year observation period, 2739 patients reported prior-month suicide attempt planning or intent and had a documented safety plan during the study period, including 745 (27.2%) who also received L2L. Over four 6-month subperiods of the observation period, L2L adoption rates increased substantially from 2% to 29% among primary care clinicians and from <1% to 48% among mental health clinicians.

**Conclusions:**

Understanding the value of L2L from users’ perspectives was essential for facilitating implementation and increasing patient reach and clinician adoption. Incorporating L2L into the existing system-level, EHR-based safety plan template reduced the effort to use L2L and was likely the most impactful implementation strategy. As rising suicide rates galvanize the urgency of prevention, the findings from this project, including L2L implementation tools and strategies, will support efforts to promote safety for suicide prevention in health care nationwide.

## Introduction

Firearm-related suicide accounts for approximately half of the suicide deaths in the United States annually [1]. Firearms are common in Americans’ lives [2]; about one-third of Americans report owning firearms [3], and an additional 10% report living in a household with a firearm [4], with higher rates in western states [2], among veterans [5], and in rural areas [6]. Moreover, the rate of ownership of new firearms appears to have increased recently among women, Black people, and Hispanic people [7]. Suicide attempts by firearm are highly lethal; researchers estimate that 85% to 95% of individuals who attempt suicide by firearm do not survive [8,9], and people with access to firearms, particularly if firearms are kept loaded and unlocked [10,11], have increased suicide risk [12,13]. Clinicians may have opportunities to intervene with patients at risk for firearm-related suicide because about 50% of individuals who die by suicide see a clinician in the month before death, and over 80% see one in the year before death [14]. Moreover, clinician-initiated discussions about reducing access to firearms have demonstrated effectiveness for improving firearm security practices (particularly in combination with free safe storage devices) [15-17], as well as promising findings for reducing suicide attempts [18,19].

Despite its potential benefits, clinician-initiated dialogue about limiting access to firearms is an uncommon practice across many primary care and mental health specialty practices [18,20]. Common barriers include time, clinicians’ unfamiliarity with firearms, and concerns about negatively impacting relationships or alienating patients [21]. “Lock to Live” (L2L) is a self-directed, anonymous, web-based decision aid that was designed to address these barriers. L2L was developed in collaboration with clinicians, firearm owners, and people who had experienced suicidal thoughts and attempts [22]. Consistent with international design standards [23,24], the L2L decision aid steps users through various considerations regarding in-home and out-of-home firearm storage options, such as types of storage, costs of storage, and background check requirements, with a goal of encouraging storage solution discussions that are consistent with the users’ values and preferences [22]. Two subsequent research studies demonstrated promising results for the feasibility and acceptability of offering L2L emergency care encounters [25] and for the uptake of L2L when it was offered via secure patient portal messages after outpatient care encounters [26]. Though L2L appears to be a useful tool for supporting suicide prevention in clinical practice, little is known about how to use L2L during routine health care encounters outside of a research context.

The goal of this project was to use mixed methods (qualitative and statistical evaluations) to support the implementation and evaluation of L2L during primary care and mental health specialty encounters in a large, regional health care system. Specifically, this project used semistructured interviews with clinicians and patients to support implementation, as well as statistical analyses to evaluate the reach and adoption of L2L over a 2-year period. The evaluation findings will inform considerations for implementing L2L nationwide to support suicide prevention in health care systems.

## Methods

### Setting

**Figure 1.**
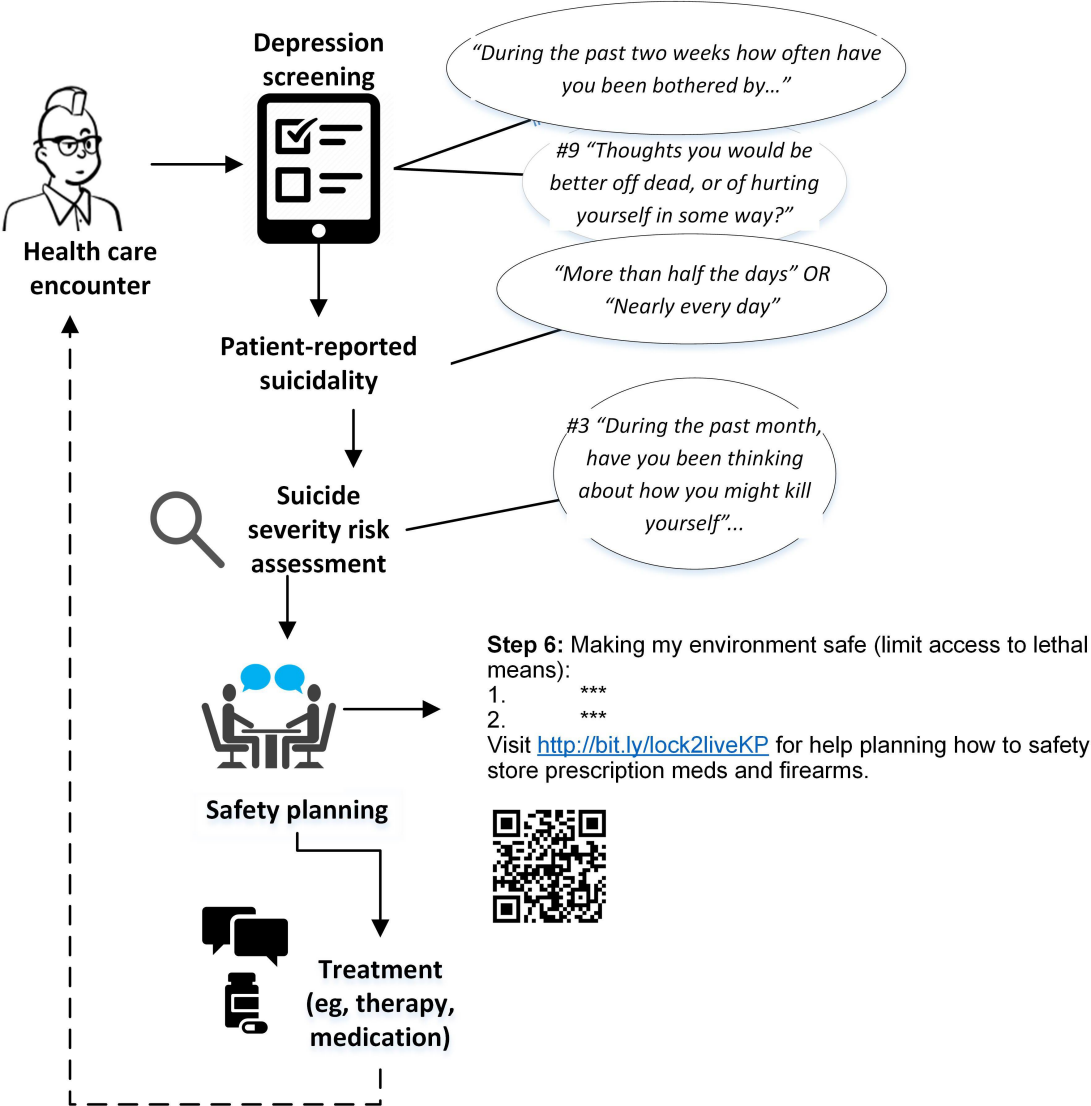
Clinical workflow for supporting the identification and engagement of patients at high risk of suicide during primary and mental health specialty encounters at Kaiser Permanente Washington.

L2L implementation and evaluation took place at Kaiser Permanente Washington (KPWA)—1 of 8 regional Kaiser Permanente health care systems, which together form one of the nation’s largest nonprofit health care organizations and serve 12.5 million people [27]. At the time of this evaluation, KPWA had provided comprehensive medical care to approximately 700,000 members across Washington State via employer-sponsored insurance plans, individual insurance plans, or capitated Medicaid or Medicare programs. In 2016, KPWA augmented standard clinical workflows to support the identification and engagement of patients at high risk of suicide attempts (Figure 1) [28,29]. Specifically, a system-level electronic health record (EHR) template was created to support clinician-initiated safety planning among patients who are identified as at high risk of suicide during primary care and mental health specialty encounters [28,30]. Nationally, safety planning is a widely recommended best practice [31], and KPWA had an established process for safety planning that included addressing access to lethal means but did not offer any specific resources to clinicians or patients about firearm storage options. Consistent with the goal of L2L, step 6 of this safety plan template was designed to support patients in limiting access to lethal means, such as firearms and prescription medications.

### Implementation and Evaluation Framework, Data Sources, and Study Design

**Figure 2.**
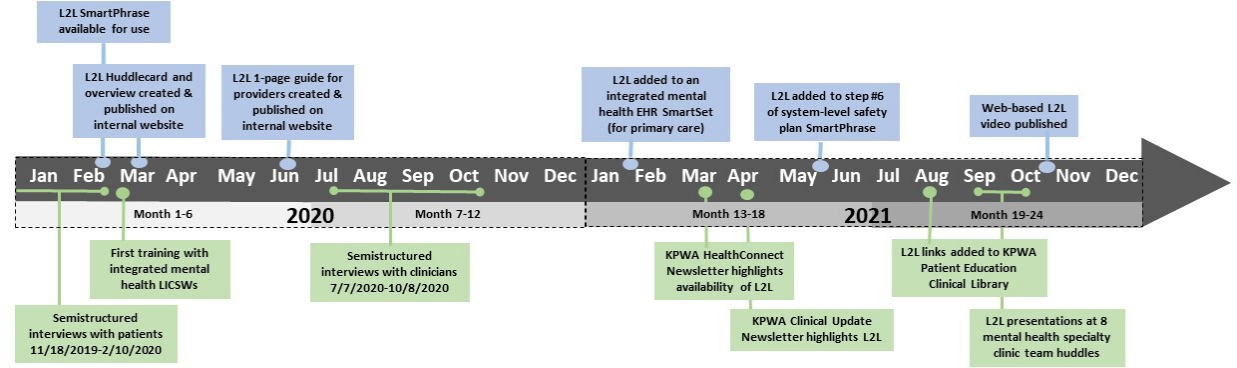
L2L implementation tools and strategies used over four 6-month subperiods of the 2-year observation period (January 1, 2020, to December 31, 2021). Tools are shown in blue, strategies are shown in green, and capped lines indicate semistructured interviews. EHR: electronic health record; KPWA: Kaiser Permanente Washington; L2L: Lock to Live; LICSW: licensed clinical social worker.

Three dimensions from the RE-AIM (Reach, Effectiveness, Adoption, Implementation, Maintenance) model*—Reach*, *Adoption*, and *Implementation* [32,33]—were selected to both inform and evaluate the implementation of L2L at KPWA (Multimedia Appendix 1) over a 2-year observation period (January 1, 2020, to December 31, 2021). Specifically, a qualitative, team-based, formative evaluation [34] was used, involving semistructured interviews with purposefully sampled patients and clinicians to facilitate implementation tools and strategies. Descriptive statistical analyses were used to evaluate the reach of L2L among patients identified at high risk of suicide and L2L adoption over a 2-year observation period. The findings were stratified by primary care and mental health specialty service settings due to the variation in the timing of the L2L trainings across these settings (Figure 2).

### Semistructured Qualitative Interviews

EHR data were used to purposefully recruit adult patients to participate in semistructured interviews that included questions to elicit suggestions about introducing L2L, as part of a broader interview that focused on exploring perceptions regarding and experiences with firearm access assessment [35]. An invitation letter was mailed to sampled patients (age≥18 y) who had received a standardized question about firearm access (“Do you have access to guns? Yes/No”) in the prior 2 weeks on a mental health questionnaire [30]. A stratified sampling distribution was used to recruit approximately equal numbers of patients in 3 groups, including those who (1) reported firearm access, (2) reported no firearm access, and (3) did not respond (ie, left the question blank). The three sampling groups were also designed to purposefully include patients who had reported thoughts about self-harm in the prior 2 weeks via the ninth question of the 9-item Patient Health Questionnaire (PHQ-9) [36]. Interviewers attempted to reach all invitees for 2 weeks to invite them to participate in a phone interview. Following the portion of the interview guide that focused on firearm access assessment [35], interviewers described how patients reporting suicidal thoughts would soon be receiving L2L and elicited feedback about how to introduce this tool in a way that would make patients more likely to use it (Multimedia Appendix 2). This portion of the interview transcript was extracted into Excel (Microsoft Corporation) and analyzed by using a team-based, rapid, qualitative inquiry approach [37]. This involved an iterative data analysis wherein 2 coders independently coded the L2L portion of the transcript by using a combination of deductive and inductive content analyses, with codes developed a priori from the interview guide as well as codes that emerged from the interviews [38]. This was followed by several rounds of discussions with 2 additional team members who reviewed the coded data and reconciled themes iteratively for the purpose of using the summarized themes to facilitate implementation.

Following the completion of the qualitative analysis that focused on patient-informed L2L implementation, interviewers initiated clinician recruitment activities, which were also more broadly focused on firearm access assessment. At the recommendation of care delivery leaders, interviewers outreached to the following two groups of clinicians responsible for engaging patients identified at risk of suicide in risk mitigation (ie, safety planning [39]): (1) licensed clinical social workers (LICSWs) supporting integrated mental health in primary care [28] and (2) consulting nurses (registered nurses) responsible for connecting patients (ie, those reporting suicidality after business hours via telephone) to telephone-based follow-up care. The L2L portion of the interview guide included questions informed by patient interviews (Multimedia Appendix 2) and was analyzed by using the same rapid, qualitative inquiry approach [37] that was used for patient interviews for the purpose of further facilitating L2L implementation.

### Descriptive Statistical Analyses

#### L2L Reach and Adoption

EHR data were used to summarize L2L “reach,” which was defined as the proportion of patients identified as at high risk of suicide via routine screening and assessment clinical workflows (Figure 1) and received the web-based decision aid. Specifically, we described characteristics of patients who had a documented safety plan during the 2-year observation period and characteristics of patients who had a safety plan that included a reference to L2L. Next, we described the adoption of L2L by primary care and mental health specialty clinicians over four 6-month subperiods of the observation period by calculating the proportions of patients identified at high risk of suicide (via suicide risk assessment; described in the *Measures* section) who had a documented safety plan with a reference to L2L and those who had a documented safety plan without a reference to L2L. We selected 6-month subperiods as the most helpful way to visually describe L2L adoption over time, since implementation paused during the initial COVID-19 outbreak (described in the *Implementation Timeline, Tools, and Strategies* section). We stratified by service setting due the variation in the timing of L2L trainings for these groups of clinicians.

#### Measures

The Columbia Suicide Severity Rating Scale (C-SSRS) was used to measure suicide risk, as per current clinical workflows. Specifically, patients reporting some level of suicide attempt planning or intent in the past month (ie, answering “yes” to C-SSRS question 3 or higher) were considered to be at “high risk” and alerted clinicians (via EHR prompts) to initiate safety planning. Distinctive phrases from standard EHR-based templates were used to detect safety plans documented in the text of clinical notes among patients identified at high risk (Multimedia Appendix 3). Sociodemographic and clinical characteristics of interest, including those known to be associated with firearm ownership and suicide risk [40,41], were measured by using the following administrative and diagnostic EHR data: age (continuous); sex (male or female); race and ethnicity (Asian, Black, Hispanic or Latinx, White, other, or unknown); insurance type (commercial, Medicare, Medicaid, or other); rurality (urban, large suburban, small suburban, or mostly rural) [42,43]; and prior year mental health, substance use, and self-harm diagnoses derived from the *International Classification of Diseases, Tenth Revision, Clinical Modification*. Reported firearm access was measured based on a positive response to the question on the mental health questionnaire [30] that was also used for qualitative interview recruitment (described in the *Semistructured Qualitative Interviews* section).

### Ethical Considerations

The project team received approval from the KPWA Region Institutional Review Board (review number: 1826198) to conduct this study. Patients who agreed to participate in the phone interview provided oral consent, including permission for the interview to be audio-recorded and professionally transcribed, and they received a US $50 cash incentive for participation. During clinician recruitment activities, clinicians received up to 3 email invitations, which included a study information sheet and instructions for opting out of participation and further contact. Participating clinicians verbally consented to participation and received a US $50 gift card for participation.

## Results

### Implementation Timeline, Tools, and Strategies

Over the 2-year implementation period (January 1, 2020, to December 31, 2021), a team of researchers and care delivery leaders took a pragmatic approach to iteratively creating and refining L2L implementation tools and strategies for primary care and mental health specialty clinicians (Figure 2). Initially, tools included an EHR-based macro (ie, EPIC SmartPhrase [Epic Systems Corporation]) for clinicians to easily add a URL and QR code referencing L2L to safety planning templates and a 1-page quick-start guide (ie, “Huddlecard”) with information on how to use the new SmartPhrase during routine clinic meetings (ie, “huddles”). In February 2020, the LICSWs who supported mental health care delivery in primary care [28] received information about L2L during a brief, web-based staff training session. Additional trainings that were planned for mental health specialty clinicians were put on hold during the widespread service disruption that subsequently occurred in response to the initial COVID-19 outbreak in March 2020. Additional tools and strategies were used, following recommendations from the care delivery leaders responsible for primary care and mental health service recovery and from the patients and clinicians who participated in semistructured qualitative interviews (detailed in the *Findings From Semistructured Qualitative Interviews* section).

### Findings From Semistructured Qualitative Interviews

Of 76 patients who were purposefully sampled during 2 waves of recruitment, 36 were interviewed from November 18, 2019, to February 10, 2020 (Table 1). Five organizing themes were derived from the portion of the interview that elicited perceptions and suggestions about L2L and were used to create a handout for clinicians, with suggestions about how to introduce L2L to their patients at risk of suicide (Multimedia Appendix 4), including recommendations to (1) “have an open conversation,” (2) “validate their situation,” (3) “share what to expect,” (4) “make it accessible and memorable,” and (5) “walk through the tool” (Table 2). In addition to these recommendations, patients expressed a preference for receiving information about L2L from “trusted” and “caring” clinicians.

**Table 1.** Characteristics of patient (n=36) and clinician (n=30) semistructured interview participants.

		Patients		Clinicians	
**Sex, n (%)**					
	Female	17 (47)		24 (80)	
	Male	19 (53)		6 (20)	
Age (y), mean (SD)		47.3 (17.9)		44.3 (12.1)	
**Age category (y), n (%)**					
	19-29	8 (22)		1 (3)	
	30-49	11 (31)		20 (67)	
	50-64	9 (25)		6 (20)	
	≥65	8 (22)		3 (10)	
**Race and ethnicity, n (%)**					
	American Indian or Alaska Native	0 (0)		1 (3)	
	Black	3 (8)		2 (7)	
	Asian or Pacific Islander	3 (8)		5 (17)	
	Latinx or Hispanic	1 (3)		4 (13)	
	Unknown	2 (6)		0 (0)	
	White	27 (75)		18 (60)	
Reported firearm access^a^^,^^b^, n (%)		16 (44)		N/A^c^	
Reported thoughts about self-harm (prior 2 wk)^a^^,^^d^, n (%)		15 (42)		N/A	

a Patients’ responses recorded on the Kaiser Permanente Washington mental health monitoring questionnaire used for criterion sampling within the 2 wk prior to the recruitment initiation.

b “Do you have access to guns? Yes/No.”

c N/A: not applicable.

d Ninth question on the 9-item Patient Health Questionnaire: “Thoughts that you would be better off dead, or of hurting yourself.”

**Table 2.** Thematic analysis of semistructured interviews with patients (n=36) and recommendations for introducing “Lock to Live” (L2L).

Themes	Illustrative quotes^a^	Recommendation
Show caring and compassion, ask permission, and respect autonomy	“I think it’s important to just take a breath, sit down with them, hold their hand, look them in the eye - ‘how can I help you? Help me help you. What’s going on? Tell me. What are you thinking? How are you feeling? How can I help you?’ Instead of an assembly line and ‘I only have a few minutes,’ so they [providers] don’t take the time.” (Patient B029) “I would hope the provider is very warm and caring and explains it’s a safety precaution, it’s for your better health and it insures you’ll be safer…basically it’s another part of your little toolbox to keep yourself well.” (Patient A032) “Probably compassionately, potentially generalized to begin with, to find out if the person is resistant right upfront… Maybe you could put a question, ‘would you be willing to consider options for storing or access to lethal means, whether it’s firearms or medication? Is it something you would be willing to discuss and look into if you were experiencing suicidal thoughts?’” (Patient A005) “We’re offering you the means to protect yourself, this is not an us decision, this is a you decision. So here is the website, the online information and we encourage you to look at it, but it’s your decision….Nobody can force you to do things. So bringing it up more as like – not we’re taking it [firearm] away from you, but letting you decide what to do with it.” (Patient A024) “Reassuring people that their firearm ownership will not end because they’re going through a rough patch in life; their ability to have their own authority to hold onto their possessions [will not end], firearms or not.” (Patient B036)	“Have an open conversation”: patients were more willing to listen and try a tool if a clinician took the time to connect, showed compassion for people’s unique experiences, and showed respect for autonomy.
Frame as helpful resource and normalize experiences	“I think overall education about the topic to start out with, just to say…‘this is what we have found is helpful, in these situations.’ Moving more into letting people know what their resources could be. Just education to be begin with, so it’s not so threatening. I think anytime somebody’s in a vulnerable place emotionally, they’re already possibly feeling threatened and they may not want to trust a lot of people.” (Patient A005) “I would hope it would be pretty real, like a conversation…‘based on your health concerns you’re showing, we’ve got some important information we’d like to share with you,’ especially if that person has a relationship or feels responsible with the person presenting it, would stay there together and talk about it afterwards….Also statistics to help a person realize how more common this is.” (Patient A011)	“Validate their situation”: normalize their experience, share how common suicidal thoughts are, and be nonjudgmental in your approach; people have a variety of gun beliefs.
Address privacy and security	“[The provider] would have to explain what it does, how it’s going to work and how private it is - nobody can get into your part of the website anyway, your personal page, where you go. So she has to reassure them about that….I just don’t feel that being online is that secure.” (Patient B004) “privacy is probably number one and an assurance that you’re not being turned in….I would be concerned in our surveillance state that disclosing things to a website about my firearm use might somehow come close to violating some kind of civil right to privacy.” (Patient B008) “As long as people don’t have to put in information which can be tracked, I can see lots of people using it. I mean the minute [you have to enter] your name, address, phone number, medical ID number, whatever else, people are going to go – eh.” (Patient A033)	“Share what to expect”: address privacy and how information is stored if patients visit the website; assure patients that L2L is anonymous.
Accessibility is key	“You don’t want to make it hard to find on a website because it doesn’t take me very long. If something’s really hard to find on a website, I’m out of there.” (Patient B004) “If there are hoops to jump through before you can access it, if you have to log in, go through a bunch of pages - maybe if it was right there, ready to access at any time, I’d say that’d be better.” (Patient B008) “I’d love to see it everywhere. Have little cards that could be given out, a billboard, having my doctor [send it].” (Patient A033) “If I knew it existed, I would probably try it. advertise it.” (Patient A032) “Highlight it in your After Visit Summary too.” (Patient A002)	“Make it accessible and memorable”: have multiple routes for sharing the website and sending reminders (after-visit summary, message, website, pamphlet).
Demonstrate and “show, don’t just tell”	“I think when you’re in a pit of despair, to go and do it on your own, some people will do that and other people will not. They need to be taken by the hand and go, ‘what do you think about this?’ Read it together.” (Patient B036) “I’m more keen to follow somebody who’s like ‘I’m offering you the opportunity to maybe do this together,’ instead of ‘I’m watching out for you.’” (Patient A024) “Being shown an example would be nice, showing it off briefly. Knowing more specifically what it does or how it could be helpful as opposed to just knowing it exists.” (Patient B033) “I think showing the patient or at least offering, would you like me to show you? Not just telling somebody, because short term memory is only like 30 s or a couple minutes and then you forget about it.” (Patient A002)	“Walk through the tool”: most patients said that a website walk-through, rather than simply having a conversation, would be helpful to overcome the barrier of trying something new, especially if already depressed.

a Identifier “A”: patients in the first wave of interviews; identifier “B”: patients in the second wave of interviews (grammatic edits, noted in brackets, were added to clarify intended meaning).

Of 51 purposefully sampled clinicians responsible for safety planning with patients identified at high risk of suicide, 30 were interviewed from July 7, 2020, to October 8, 2020 (Table 1), including 25 LICSWs and 5 registered nurses. During the interviews with LICSWs, only 3 had actually used L2L with a patient—9 were unfamiliar with L2L, and 12 were familiar with but had not yet used L2L. Most clinicians saw clear benefits to L2L as an option for supporting both clinicians and patients. Several clinicians expressed concern about using the tool to replace dialogue about lethal means, and most supported the idea of a walk-through, as patients had recommended. Clinicians also expressed a strong preference to have L2L information included by default in the EHR-based safety planning template, in contrast to having clinicians remember to add it (via SmartPhrase). A clinician also suggested automatically including L2L in after-visit summaries when patients reported thoughts about self-harm on the PHQ-9. The implementation team worked with clinical partners to update the system-level, EHR-based safety plan template to include L2L information and updated the Huddlecard to communicate this change (Multimedia Appendix 5). After-visit summaries were used to provide safety plans to patients who were seen via a secure, web-based patient portal, and L2L was automatically included after the template change. Several clinicians also requested follow-up trainings or refreshers about L2L. The team therefore conducted a round of brief trainings, which were presented during routine clinic huddles with mental health specialty clinicians, and created a 3-minute training video (Multimedia Appendix 6).

### Findings From Descriptive Statistical Analyses

During the study period, 2739 adult patients reported some prior-month suicide attempt planning or intent via routine suicide risk assessment workflows during primary care or mental health specialty encounters and had a documented safety plan, including 745 (27.2%) who also received L2L. Overall, there were no major differences in the demographic and clinical characteristics between patients who received L2L and the broader population that was identified as at risk of suicide and had a documented safety plan (Table 3).

**Table 3.** Characteristics of patients who received “Lock to Live” (L2L; n=745) and were among patients with a documented safety plan (n=2739) during the implementation period (January 1, 2020, to December 31, 2021).

		Patients who received L2L, n (%)		Patients with a documented safety plan^a^, n (%)	
**Age^b^** **(y)**					
	18-39	513 (68.9)		1817 (66.3)	
	40-64	187 (25.1)		732 (26.7)	
	≥65	45 (6)		190 (6.9)	
**Sex^c^**					
	Female	445 (59.7)		1753 (64)	
	Male	300 (40.3)		986 (36)	
**Race and ethnicity^c^**					
	American Indian or Alaska Native	14 (1.9)		75 (2.7)	
	Asian	54 (7.2)		199 (7.3)	
	Black	52 (7)		166 (6.1)	
	Hawaiian or Pacific Islander	8 (1.1)		45 (1.6)	
	Hispanic or Latinx	59 (7.9)		201 (7.3)	
	Unknown	93 (12.5)		294 (10.7)	
	White	465 (62.4)		1759 (64.2)	
**Insurance^c^**					
	Commercial	530 (71.1)		1831 (66.8)	
	Medicare	83 (11.1)		342 (12.5)	
	Medicaid	54 (7.2)		228 (8.3)	
	Not enrolled	78 (10.5)		338 (12.3)	
**Rural or urban** ^**c**^ **^,^ ^d^**					
	Urban	301 (40.4)		1010 (36.9)	
	Large suburban	205 (27.5)		799 (29.2)	
	Smaller suburban	186 (25)		802 (29.3)	
	Mostly rural	31 (4.2)		96 (3.5)	
**Mental health diagnoses^e^**					
	Depression	675 (90.6)		2502 (91.3)	
	Anxiety	652 (87.5)		2434 (88.9)	
	Serious mental illness	144 (19.3)		586 (21.4)	
	Substance use disorder	196 (26.3)		752 (27.5)	
	Suicide attempt diagnosis	39 (5.2)		175 (6.4)	
Reported firearm access^e^		150 (20.1)		501 (18.3)	

a Includes safety plans with L2L.

b At evaluation midpoint (January 1, 2021).

c At first encounter.

d Missing information for 22 patients.

e During implementation period.

The adoption of L2L increased substantially over the 2-year observation period (Tables 4 and 5). During this time, rates of documented safety plans among patients identified at high risk of suicide (C-SSRS score≥3) remained fairly consistent—51.2% to 55.2% of primary care patients and 73.4% to 78.4% of mental health specialty patients had a documented safety plan. However, over four 6-month subperiods of the observation period, L2L adoption rates increased substantially from 2% to 29% among primary care clinicians and <1% to 48% among mental health clinicians, increasing primarily after L2L was integrated into the EHR-based safety planning template.

**Table 4.** Proportions of primary care patients who were identified as at high risk of suicide and had a documented safety plan during primary care encounters over the implementation period (January 1, 2020, to December 31, 2021).

Subperiods of implementation period	Patients with a documented safety plan that did not include “Lock to Live,” %	Patients with a documented safety plan that did include “Lock to Live,” %
Months 1-6	53.5	1.6
Months 7-12	50	4.1
Months 13-18	41.7	11.1
Months 19-24	22.2	29.1

**Table 5.** Proportions of primary care patients who were identified as at high risk of suicide and had a documented safety plan during mental health specialty encounters over the implementation period (January 1, 2020, to December 31, 2021).

Subperiods of implementation period	Patients with a documented safety plan that did not include “Lock to Live,” %	Patients with a documented safety plan that did include “Lock to Live,” %
Months 1-6	78.1	0.3
Months 7-12	74.8	1.4
Months 13-18	53.8	19.6
Months 19-24	25.9	48.4

## Discussion

This novel study used mixed methods to support the implementation and evaluation of a web-based decision aid that was designed to help patients at risk of suicide limit access to firearms. Specifically, findings from semistructured interviews with patients and clinicians were used to facilitate L2L implementation, while statistical analyses were used to describe rates of reach among patients identified at risk of suicide and increased adoption by clinicians who cared for them during the 2-year observation period.

L2L development centered users’ values and preferences in the design process [22]. Similarly, the tools and strategies developed for this project used information from semistructured interviews with people who were the most likely to be impacted by L2L implementation, including firearm owners, patients experiencing suicidality, and the clinicians who care for them. Clinicians have reported a lack of experience with handling firearms and have expressed apprehension about discussing firearm safety due to concerns about damaging relationships with patients [44-46]. Likewise, patients have expressed apprehension about disclosing access to firearms due to concerns about privacy, autonomy, and firearm ownership rights [47,48]. For these reasons, patients and clinicians perceive firearm access assessment as challenging but also as valuable for supporting suicide prevention [35]. This implementation project showed that clinicians, that is, those responsible for engaging at-risk primary care and mental health patients in suicide risk mitigation, willingly adopted the use of L2L to support safety planning.

This study also has important implementation implications. Unsurprisingly, the rates of L2L adoption increased after L2L was incorporated into the existing system-level safety planning template as a default (primarily in the latter half of year 2). This finding underscores the importance of removing barriers to the adoption of web-based decision aids and making adoption “easy” [49]. In contrast, those seeking change often focus on amplifying benefits or “selling” their new idea or innovation; however, it may be equally as important or more important to focus on “friction,” that is, “psychological forces that oppose and undermine change,” such as inertia, effort, emotion, and reactance [50]. In the case of L2L, reducing the effort required for clinicians to remember to use L2L appeared to be the main driver of its adoption. However, the tools and strategies that were designed to communicate about the benefits of using L2L (eg, training, video, Huddlecard, and newsletter information) were likely necessary for leaders to understand L2L’s value to patients and clinicians and approve the system-level change that was required to make L2L easier to use for clinicians.

This study has important clinical implications for supporting suicide prevention in health care. First, L2L supports clinicians who engage patients identified at risk of suicide in collaborative safety planning and lethal means counseling, which are evidence-based suicide risk mitigation practices that are recommended by the Zero Suicide Institute and follow the principles outlined in the National Strategy for Suicide Prevention [29,51-53]. Moreover, the recommendations from interview participants (“have an open conversation,” “share what to expect,” and “walk through the tool”) support a motivational interviewing approach to lethal means counseling and align with the recommendations of the Veterans Health Administration [54]. Second, L2L was developed by patients with lived experiences of suicidality and firearm ownership; therefore, L2L supports cultural competency in health care as a culturally aligned intervention [55]. Finally, this technology-based, EHR-embedded approach to addressing lethal means supports all 6 aims of health care quality that are outlined by the Institute of Medicine—*safe*, *effective*, *patient-centered*, *timely*, *efficient*, and *equitable* [56,57].

There are several limitations of this project that have implications for future research. First, the implementation of L2L at KPWA occurred during the initial outbreak of a global pandemic, which impacted the original implementation plans while health care systems responded to the pandemic and rapidly shifted toward providing web-based mental health care [58]. Semistructured interviews with patients took place prior to this shift. Future research should explore optimizing mental health care delivery workflows that support web-based suicide risk identification (ie, screening and assessment) [59] and incorporating L2L in web-based care encounters via secure telehealth platforms that are designed to support patient engagement. Second, L2L recognizes and addresses firearm policies related to background checks and how these policies might influence the legality of temporary firearm transfers for addressing suicide risk, but it does not address specific state laws. Additional work to understand the legality of recommendations about firearm safety practices may be helpful for health care systems that implement L2L. Third, this project was not designed to measure the specific impact of individual implementation strategies or determine whether L2L was effective in helping patients reduce access to firearms for suicide prevention purposes. Measuring the effectiveness of this tool, which was designed to support population-based suicide prevention, would require extending the implementation of L2L to other large health care systems nationwide and conducting other analyses that are designed to measure key functions of suicide prevention practices, including risk identification, engagement in evidence-based risk mitigation and treatment, and supportive care transitions [29]. Finally, L2L is meant to support adult patients at risk of suicide reduce access to firearms and other lethal means; additional tools and strategies are required to support youth at risk of suicide. Notably, there is a similar web-based decision aid that is available for this purpose; “Lock and Protect” was designed to help parents and caregivers reduce access to lethal means for youth suicide risk mitigation [60]. Similarly, the “Safety in Dementia” web-based decision aid was developed to support caregivers in addressing firearm access among individuals with Alzheimer disease and related dementias [61]. Future research should evaluate the implementation of these tools in routine care delivery.

In conclusion, incorporating L2L into the existing system-level safety plan template reduced the effort required to use L2L and was likely the most impactful implementation strategy for increasing clinician adoption and patient reach. However, understanding the value of L2L from the users’ perspectives was essential for effectively amplifying the suicide risk mitigation benefits. As rising suicide rates galvanize the urgency of prevention [62], the implementation tools and strategies developed for this project will be useful for health care systems nationwide.

## Supplementary material

10.2196/48007 Multimedia Appendix 1 The three dimensions of RE-AIM (Reach, Effectiveness, Adoption, Implementation, Maintenance) selected to inform and evaluate the implementation of “Lock to Live.”

10.2196/48007 Multimedia Appendix 2 Patient and clinician interview questions that focused on “Lock to Live” implementation.

10.2196/48007 Multimedia Appendix 3 Elements included in the search for safety plans documented in clinical notes text.

10.2196/48007 Multimedia Appendix 4 Introducing Lock2Live.org: a guide for clinicians.

10.2196/48007 Multimedia Appendix 5 “Lock to Live” Huddlecard.

10.2196/48007 Multimedia Appendix 6 “Lock to Live” video for Kaiser Permanente Washington clinicians.

## Abbreviations

C-SSRS: Columbia Suicide Severity Rating Scale
EHR: electronic health record
KPWA: Kaiser Permanente Washington
L2L: Lock to Live
LICSW: licensed clinical social worker
PHQ-9: 9-item Patient Health Questionnaire
RE-AIM: Reach, Effectiveness, Adoption, Implementation, Maintenance
