# Supplementary material for: Reducing Firearm Access for Suicide Prevention: Implementation Evaluation of the Web-Based “Lock to Live” Decision Aid in Routine Health Care Encounters
Source: JMIR Med Inform. 2024 Apr 22;12:e48007. doi: 10.2196/48007 (PMC11063417; doi:10.2196/48007)
Supplement: Multimedia Appendix 1 [file medinform-v12-e48007-s001.docx]

Multimedia Appendix 1. Three dimensions of RE-AIM selected to inform and evaluate implementation of L2L.

| **RE-AIM Domain** | **Research Question(s)** | **Data-Collection Source & Study Design** |
| --- | --- | --- |
| **Reach** | What proportion of patient visits with a documented safety plan included provision of L2L? How did patients who received L2L compare to those who did not receive L2L? | Statistical analysis of electronic medical record data |
| **Adoption** | How did L2L provision vary over time in target care settings (i.e., primary care and mental health specialty)? | Statistical analysis of electronic medical record data & qualitative documentation of implementation timeline, tools, and strategies. |
| **Implementation** | How did patients recommend introducing L2L and facilitating use by patients at risk of suicide?  What were provider perceptions of L2L and recommendations for facilitating use by providers and patients? | Formative qualitative evaluation via semi-structured interviews with purposefully sampled key informants. |
