# Supplementary material for: Reducing Firearm Access for Suicide Prevention: Implementation Evaluation of the Web-Based “Lock to Live” Decision Aid in Routine Health Care Encounters
Source: JMIR Med Inform. 2024 Apr 22;12:e48007. doi: 10.2196/48007 (PMC11063417; doi:10.2196/48007)
Supplement: Multimedia Appendix 2 [file medinform-v12-e48007-s002.docx]

Multimedia Appendix 2. Patient & Clinician Interview Questions Focused on L2L Implementation.

**Patient Interview Guide Excerpt:**

We are hoping to get your feedback about a new web-based resource we’ll be giving patients. You may be able to answer based on personal experience but answering hypothetically is ok too.

- 1. When patients report frequent suicidal thoughts, providers will provide an anonymous online tool to help them make decisions about lethal means safety, like storing medications and firearms. What do you think providers could do to make this interaction feel safe and comfortable for the patient?
  2. Based on your experience or hypothetically, how would you want to hear about this tool if you were thinking about suicide and had access to firearms?
  3. How would you want your provider to start this conversation?
  4. What would maybe make you more likely to try out the web-based tool?

**Provider Interview Guide Excerpt:**

We are hoping to get your feedback about a website that guides patients through a series of questions and concludes with personalized recommendations for safe storage of firearms. Clinicians and patients, including firearm owners and those with suicidal thoughts helped develop this web-based decision aid called Lock2Live. Providers can now enter a standard .dot phrase *.LOCK2LIVE* to add a short URL and a QR code for easy access to this resource in a crisis response plan or after-visit summary.

- 1. What are your thoughts about this decision aid? (Probe if needed: provider experiences with L2L)
  2. We talked to patients about their preferences, some told us a simple link in a secure message would be fine, but others said they would be a lot more likely to try Lock2Live if their providers tried it with them first particularly in cases when they were feeling really depressed and suicidal. What are your thoughts about how providers could encourage their patients with firearm access and suicide risk to use Lock2Live?
  3. What tools or prompts might be useful for helping you introduce Lock2Live and encourage patients to try it? Would automatically populating the crisis response plan template with a URL and QR code helpful? What other ideas do you have?
  4. What kind of follow-up do you think might be helpful after the introduction of Lock2Live? Would some kind of prompt to check-in with patients at risk of suicide be helpful? Do you have tools you utilize now for that follow-up?
