# Supplementary material for: Reducing Firearm Access for Suicide Prevention: Implementation Evaluation of the Web-Based “Lock to Live” Decision Aid in Routine Health Care Encounters
Source: JMIR Med Inform. 2024 Apr 22;12:e48007. doi: 10.2196/48007 (PMC11063417; doi:10.2196/48007)
Supplement: Multimedia Appendix 3 [file medinform-v12-e48007-s003.docx]

**Multimedia Appendix 3. Elements included in search for safety plans documented in clinical notes text.**

| ***Safety Plan Elements** | **KPWA SmartPhrase element (adult version)** | **KPWA SmartPhrase element**  **(teen version)** | **Search phrases**  **(case-insensitive)** |
| --- | --- | --- | --- |
| **Step 1: My early warning signs (thoughts, images, mood, situation, behavior) that a crisis may be developing:** | My early warning signs that I am in crisis: *** | Early warning signs (thoughts, images, mood, behaviors) that I am headed to a crisis: | -warning signs |
| **Step 2: My internal coping strategies - Things I can do to take my mind off my problems without contacting another person (relaxation technique, physical activity):** | Things I can do and skills I can practice to help me feel better: *** | Things I can do to stay safe and take my mind off of the problem: {Options to help me:22560} (additional suggestions are deep breathing,or watching a funny video) | -Things I can do  -Things that I can do  -skills I can practice  - skills I should practice  -I will try the following skills  -coping strategies  - coping techniques |
|  | Things to avoid which make me feel worse: *** | Triggers (things to avoid which make me feel worse): *** |  |
| **Step 3: My external strategies with people and social settings that provide a healthy distraction:** |  | People and places that provide distraction: (examples are a friend, or your room).*** |  |
|  |  | Things my parents and others who care about me can do when they notice my warning signs: *** | [detected by “warning signs” or “my parent or others who care”] |
| **Step 4: People whom I can ask for help during a crisis:** | Support person(s) I can contact (include phone number): *** | Adults I can ask for help: (examples are a guidance counselor, or family member) *** | -support person  -support people  - supportive contacts  -I can ask for help  -my parents and others who care  -I will call the following people(kpco)  -who can I contact?  - Supportive family members or friends |
| **Step 5: Professionals or clinics I can contact during a crisis:** | Contact info for resources: {Primary Care Team:22563}  {BHS Specialty Clinic:22564}  The Suicide Lifeline Crisis Line 1 800 273 TALK (8255)  {County Crisis Line:22566} | Contact info for resources:  To reach your Adolescent Center health care provider call 425-562-1350  {Primary Care Team:22563}  {BHS Specialty Clinic:22564}  The Suicide Lifeline Crisis Line 1 800 273 TALK (8255)  {County Crisis Line:22566}  Call the Kaiser Permanente Consulting Nurses at 206-901-2244 and tell them you are a patient at the Adolescent Center. | -Lifeline  -1-800-273-TALK  -1-800-273-(TALK) 8255  -1-800-273-(TALK)8255  -273-8255  -273.8255  -Crisis Line  -Crisis text  -Crisis chat  -Crisis clinic  -741741  -I can contact during a crisis  - Nacional de Prevención del Suicidio: 1-888-628-9454  - Línea de Crisis  -Trevor Project  -trevorproject  -1-866-488-7386 |
|  |  | Crisis text line: text 741741 anytime and a live trained crisis counselor receives the text and responds quickly to help you move from a hot moment to a cool calm one to stay safe using active listening and suggested referrals through secure messaging |  |
| **Step 6: Making my environment safe (limit access to lethal means):** | Things I can do to limit my access to lethal means: *** | Things to make my environment safe (and limit access to lethal means)*** | -my environment safe  -ensure that my environment is safe  -to lethal means  -have you stockpiled any medications  -that I have access to that  are related to methods I am considering  - lock2live  - LockToLive |
| **Optional Steps** |  | Something that is important to me and worth living for is: *** |  |
|  |  | Things that I can do to take good care of myself: (examples are getting enough sleep, regular meals, and regular exercise): *** |  |
| **Additional Directions (KPWA specific)** | The best number my provider can call me at is: *** | The best number my provider can call me at is: *** |  |
|  | If I cannot be reached, my provider can call *** (emergency contact) | If I cannot be reached, my provider can call (emergency contact) *** |  |
|  | If I cannot keep myself safe, I will go to the nearest emergency room or call 911 | If I cannot keep myself safe, I will go to the nearest emergency room or call 911 |  |
|  |  | My next appointment is: *** |  |
| **Labels** | Crisis Response Plan | Safety Plan | -Crisis Response Plan  -Crisis Plan  -Safety Plan |
| **Must include 2 steps to count as documented safety plan.* | | | |
