# Supplementary material for: Reducing Firearm Access for Suicide Prevention: Implementation Evaluation of the Web-Based “Lock to Live” Decision Aid in Routine Health Care Encounters
Source: JMIR Med Inform. 2024 Apr 22;12:e48007. doi: 10.2196/48007 (PMC11063417; doi:10.2196/48007)
Supplement: Multimedia Appendix 4 [file medinform-v12-e48007-s004.docx]

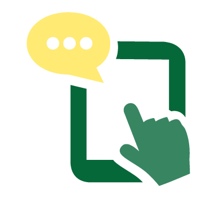

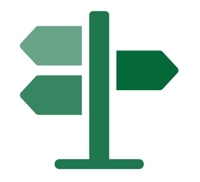

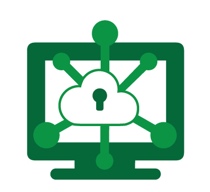

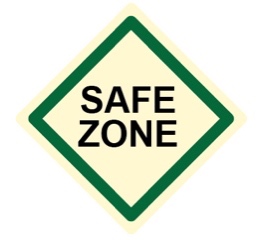

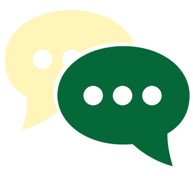


**Kaiser Permanente member voices:**  what would make them more likely to try Lock2Live.org:

- Online anonymous decision tool to support patients at risk for suicide
- Resources for temporary storage of firearms and other dangerous household items

# Introducing Lock2Live.org A guide for providers

# **Have an open conversation**

How you introduce Lock2Live matters. Patients were more willing to listen and try a tool if a provider took the time to **connect**, showed **compassion** for people’s unique experiences, and showed **respect for autonomy.**

# **Validate their situation**

**Normalize their experience** by sharing how common suicidal thoughts are and **be nonjudgmental** in your approach; people having a wide variety of gun beliefs.

# **Share what to expect**

**Describe the tool**: what it does and how long it takes. **Address privacy** and how information is stored if they visit the website.  Assure them that L2L is anonymous.

# **Make it accessible and memorable**

Have **multiple routes** for sharing the website and sending reminders (in person, secure message, after visit summary, website, pamphlet).

# **Walk through the tool**

Patients said a website **walk- through** was useful.  Many felt **a trusted provider showing them** was necessary, in order to overcome the barrier of trying something new, especially if already depressed.

***“I think it's important to just take a breath, sit down with them, look them in the eye - how can I help you? What's going on? How are you feeling?”***

***“So bringing it up more as like – not we’re taking it [firearm] away from you, but letting you decide what to do with it….I’m more keen to follow somebody who’s like ‘I’m offering you the opportunity to maybe do this together,’ instead of ‘I’m watching out for you.’”***

***“Explain what it does, how it's going to work and how private it is, that nobody can get into your data.”***

***“Maybe the first time have the provider show me. If I'm suicidal, I'm probably not going to be… looking for things on my own.”***
