# Supplementary material for: Reducing Firearm Access for Suicide Prevention: Implementation Evaluation of the Web-Based “Lock to Live” Decision Aid in Routine Health Care Encounters
Source: JMIR Med Inform. 2024 Apr 22;12:e48007. doi: 10.2196/48007 (PMC11063417; doi:10.2196/48007)
Supplement: Multimedia Appendix 5 [file medinform-v12-e48007-s005.docx]

**Multimedia Appendix 5. L2L Huddlecard.**


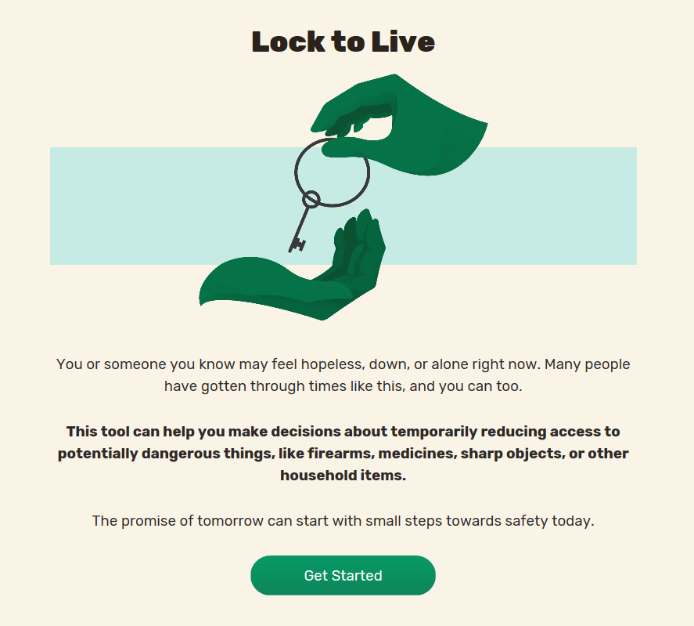

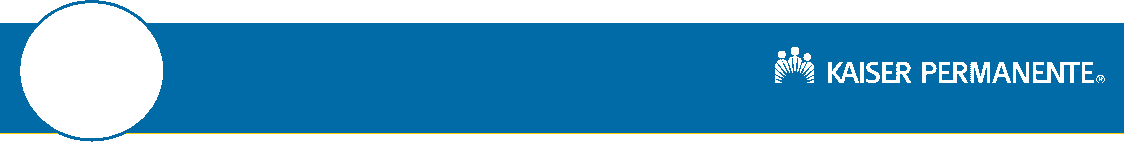


**HUDDLE CARD**

**Lock2Live Decision Aid**

**What’s Lock2Live?** A patient-facing web-based decision aid is available to help individuals at risk of suicide make decisions about lethal means safety, particularly firearms and prescription medications. Clinicians and patients (including firearm owners and those with suicidal thoughts) helped develop this tool.

**Link to 3 minute training video:** [**here**](https://vimeo.com/640536449/55bb71fe9e)

**When/How do I use Lock2Live?**


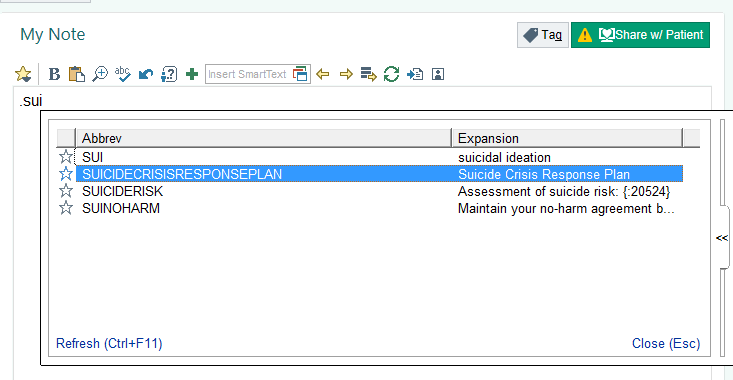
During crisis response planning (via .SUICIDECRISISRESPONSEPLAN) when: 1) patients are identified as having past-month intent or plans for suicide attempt (e.g. Columbia score ≥3),

2) when providers are seeing patients with an existing safety plan, or

*
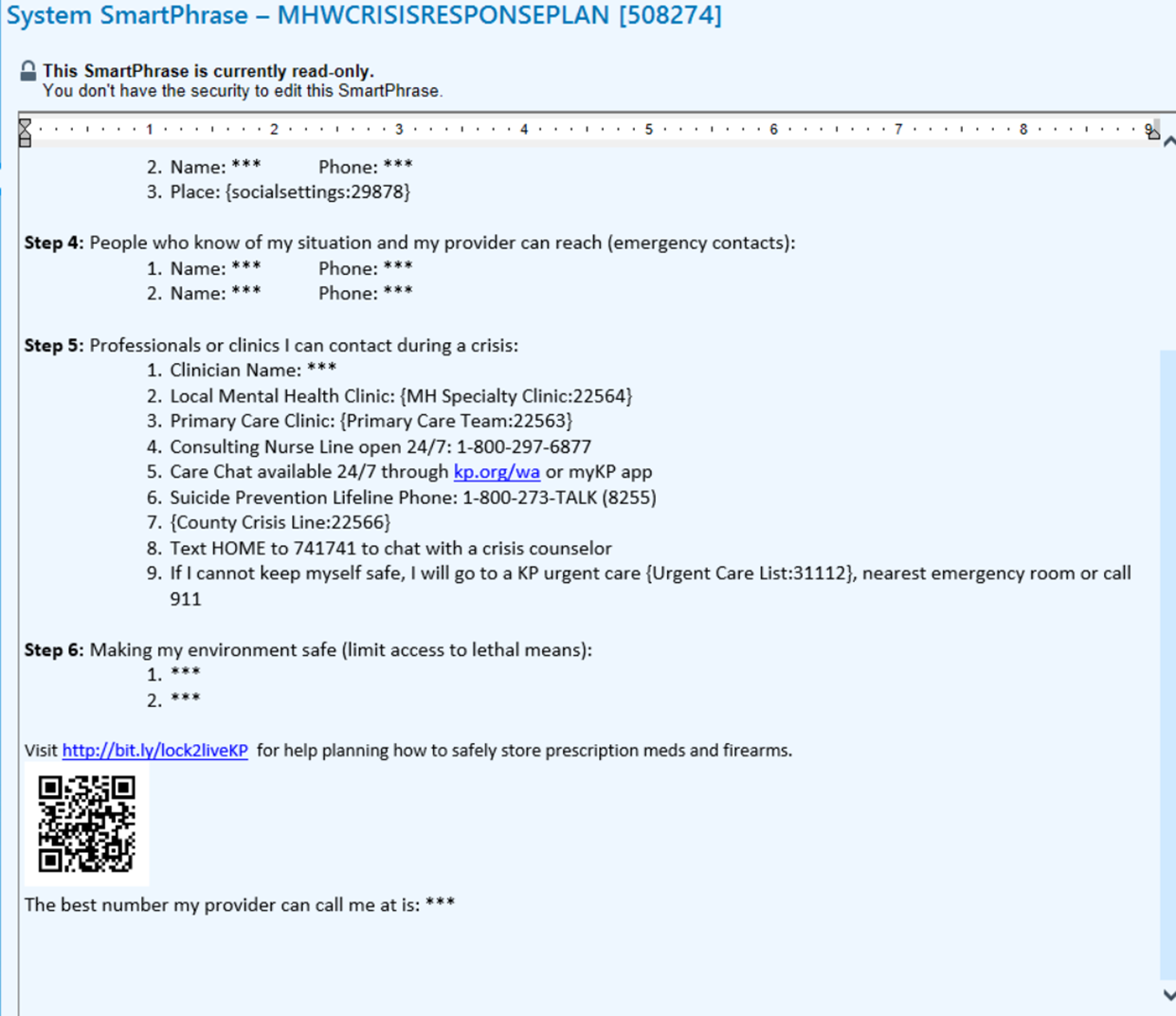
*3) at the discretion of the provider.

Providers can also use **.LOCK2LIVE**  to add a short URL <http://bit.ly/lock2liveKP> and a QR code for easy access to this resource (i.e. in AVS or Secure Message)

**
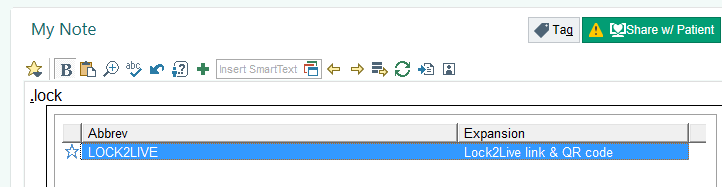
**


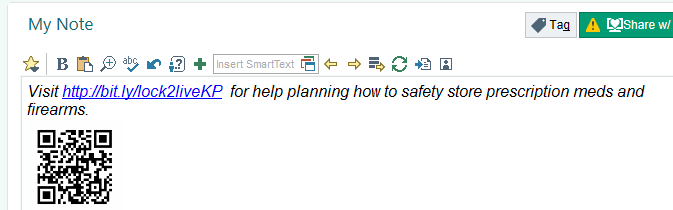


**FAQs**

**Why Use Lock2Live?** Providers may have valuable opportunities to intervene with patients at risk of suicide, and reducing accessibility of highly lethal and commonly used suicide methods is associated with declines in suicide rates.

**How should I introduce Lock2Live to patients?** Here’s some example scripting: *“You indicated earlier you have a gun in your home.  Can I show you Lock2Live on my computer /or/ your phone? It’s this really helpful tool for figuring how you can store your firearms in a way that makes it less likely you will use it when you may be experiencing intense emotional pain.”*

The shortened URL and QR code makes it easy to help you and the patient access the site up on their phone (using the camera) and doing it together first may help the patient feel comfortable using it on their own later. More info on introducing Lock2Live [here](https://sp-cloud.kp.org/sites/kpwa-bh/bh_integration/documents/lock2live.org_2020.6.24.pdf)

**What does Lock2Live look like?** Please visit [Lock2Live](http://lock2live.kpwashingtonresearch.org/) and try it out

**What else do I need to know (patients might ask about)?**

- Lock2Live is designed to augment (not replace) conversations about lethal means
- Lock2Live does not store any identifiable information
- Lock2Live is hosted on a secure internal webserver behind the KPWA firewall
- Lock2Live is a free resource & no 3^rd^ party vendors are involved
- Background checks for temporary transfers of firearms are not required in Washington State for suicide prevention purposes
